# Talking about chronic pain: Misalignment in discussions of the body, mind and social aspects in pain clinic consultations

**DOI:** 10.1177/13634593211032875

**Published:** 2021-07-22

**Authors:** Jana Declercq

**Affiliations:** University of Groningen, Netherlands

**Keywords:** chronic pain, discourse analysis, doctor-patient interaction, health communication, mind-body dualism

## Abstract

In Western societies, human existence and illness are mostly constructed from the perspective of mind-body dualism: body and mind are considered to function independently, and the body/the physical as primary and more real. Research shows, however, that mind-body dualism is no longer tenable, especially in healthcare contexts. This led to the rise the biopsychosocial model, in which bodily experiences, including illness, are seen an interplay of the physical and the psychological, and the social. This model is the current gold standard for treating chronic pain. As these perspectives on the body and illness are potentially conflicting, and discursively constructed, this paper examines whether they are a source of misalignment in interactions between chronic pain patients and their doctors in a pain clinic. The analysis shows these perspectives indeed lead to misalignment, for instance when discussing the relevance of psychotherapy, and lead to intricate uses of argumentative resources to account for the differing perspectives on (the treatment of) pain.

## Introduction

Traditionally, Western societies mostly construct human existence and bodily experiences, including illness, from a dualistic perspective, in which body and mind are seen as two separate entities functioning independently (Scheper-hughes and Lock, 1987; Slatman, 2014). This dualism is termed mind-body dualism, or Cartesian dualism, and is considered a basic foundation of medical research and practice, and several other domains in Western cultures. However, there is evidence that bodily experiences should be conceptualised as a multi-directional interplay of physical, psychological, social and cultural factors (Gatchel et al., 2007; Slatman, 2014). Consequently, this approach is increasingly installed in clinical practice.

These perspectives are inherently contradictory, which may create tensions in health care communication, when patients and doctors have different understandings of bodily experiences. This tension is especially of interest for chronic pain, as, in this context, psychological and social factors, such as stress, (lack of) social recognition, stigma, and the meanings attached to the illness, are an integral part of the illness experience, and included as factors in treatment plans (Jackson, 2005; Scheper-hughes and Lock, 1987).

While these perspectives have been researched in a health sociology, philosophy and medical research and practice, it is unclear how they discursively emerge in healthcare interactions, and whether they are a potential source of misalignment and resistance. As these perspectives are essentially discursive and constructed, negotiated and resisted in interaction, interactional analysis can advance our understanding of perspectives on bodily experiences in healthcare communication. This paper therefore analyses 13 doctor-patient interactions in a Belgian pain clinic, in which the health professionals take a multidisciplinary, biopsychosocial approach to treating pain. This paper aims to answer the following research questions:

- *Do potentially different perspectives on body, mind and social aspects lead to misalignment and resistance in chronic pain consultations?*
- *Which argumentative and interactional resources are being drawn upon by patients to discuss, negotiate and mitigate perspectives on the body in cases of misalignment and resistance?*

## Theoretical framework

### Mind-body dualism and the biopsychosocial model

Mind-body dualism has been and still is a viable and prominent perspective on human existence in Western society (Burgmer and Forstmann, 2018; Jeffries, 2007; Malson and Ussher, 1996; Paechter, 2004). As a result of the broad range of contexts in which it appears, mind-body dualism is not a unified perspective, but takes several shapes. In medical contexts, in its oldest, narrowest interpretation, mind-body dualism implies a view on the body in which all illness is caused by traceable tissue injury (Bendelow and Williams, 1995). More recently, mind-body dualism included the mind as something that can be subject to illness and health problems. In this view, however, there is a split between the body and mind: illness is either physical or psychological, in its causes, symptoms and treatment (Glew and Chapman, 2016).

Mind-body dualism comes with hierarchical perspectives on mind and body. Physical pathologies have been considered primary, objective, real and accidental, and psychological ones are seen as secondary, subjective, unreal and imagined, and intentional (Jackson, 2005; Scheper-hughes and Lock, 1987). Consequently, when mind and body are dichotomised, pathologies falling in either category are also evaluated differently socially; historically, mental health and psychological illness have been associated with more stigma (Rüsch et al., 2005).

Empirical studies have examined concrete manifestations of mind-body dualism in daily life (Gillies et al., 2004; Jeffries, 2007; Malson and Ussher, 1996; Paechter, 2004), providing evidence of the salience of a dualistic perspective in the context of bodily experiences and illness, and its consequences. For instance, mind-body dualism is associated with poorer health choices, as dualists assume that physical problems have no effect on mental health (Burgmer and Forstmann, 2018).

However, today, such a dualistic view is deemed no longer tenable, especially in healthcare contexts. Consequently, an alternative perspective rose to prominence: the biopsychosocial (BPS) model (Gatchel et al., 2007), which conceptualises bodily experiences such as illness as an interplay of the physical and the psychological, and a third dimension: the social. The BPS model was introduced by Engel in 1977 in the context of psychiatry, but was subsequently also integrated in other clinical contexts, most prominently in chronic pain care (Jull, 2017). In the model, psychological factors include emotions and stress, but also the meanings one attaches to emotional experiences, and how these meanings influence illness experiences. Social dimensions traditionally comprise environmental stressors, interpersonal relationships, work history, social expectations, and cultural factors (Engel, 1977; Glew and Chapman, 2016; Jull, 2017).

Since its introduction in the seventies, the model has been criticised, further developed, and become less and more prominent again. Critical scholars addressed the model was still dualistic and only adding, but not integrating the social; for being vague conceptually, for instance in relation to what culture is; for being imbalanced, mostly lacking attention for the social; and for being difficult to implement (Benning, 2015; Jull, 2017). Further developments, partly addressing these criticisms, have for instance further developed the social domain: more recent literature also highlights different and more specific social factors (that are often seen as intersecting and interrelated), such as social identities and locations; personal experiences related to discrimination, dismissal, violence, and stigma; and access to and quality of health care (Wallace et al., 2021). Other scholars further developed the model by propagating that the three domains are not static and equally contributing, but fluid, and their weight different for each patient (Jull, 2017), and by designing more practical guidelines on how to implement the model (e.g. Cheatle, 2016), sometimes for specific patient groups (e.g. Miaskowski et al., 2020). At the same time, the prominence of the BPS model has varied over time and in different medical domains: in the case of chronic pain, for instance, some health care professionals have gone back to a strong and sometimes sole focus on nociception; similarly, others have only focused on merely treating psychosocial features (Jull, 2017).

Now, however, in clinical practice, the consensus for several pathologies is that multidisciplinary BPS-based treatment is more effective than monodisciplinary treatments, and therefore is the gold standard of treating these pathologies, including chronic pain (Kamper et al., 2015). In what follows, I will explore the relevance and use of the BPS model in pain treatment in more depth.

### Chronic pain

Chronic pain is pain that lasts longer than expected for a particular injury, and affects daily life and wellbeing (Berquin et al., 2011). Overall 8.5% of Belgian citizens seek help for chronic pain in a pain clinic; as not all patients seek specialised help, the actual number of chronic pain patients is likely higher (Berquin et al., 2011); worldwide, prevalence is about 10% (Andrews et al., 2018).

Chronic pain is difficult to treat, and living with it comes with great challenges. Often, finding a clear somatic cause is not possible, which can be challenging to accept for patients and their environment. Chronic pain patients often struggle with not being believed and recognised, and feeling stigmatised (Jackson, 2005). Most importantly, research indicates, when considering research on clinical practice on both patients’ and providers’ perspectives, implementation of the BPS model in the context of chronic pain can be challenging (Darnall et al., 2017). Dwyer et al. (2017) list a number of factors and barriers making it difficult to implement the BPS model consistently when judging chronic pain in primary care, such as GP attitudes, time, patient-doctor relationship, and patient perceptions. This latter factor is also found to be crucial by Purcell et al. (2019), who show that patients’ experiences with BPS-modelled care vary widely, depending on whether patients are willing to reduce medication intake and what their expectations of the treatment plan are, and what their opinions are on the value of behavioural and integrative treatment. Kenny (2004) similarly illustrates that doctor-patient interactions on pain often contain an implicit dialogue in which doctors negotiate the importance of psychological dimensions, while patients often put (solely) somatic causes at the forefront. Communication thus is key, as will be developed more in the next section.

### The importance of language

In negotiating understandings of the body, illness, pain, and treatment, language is essential in several ways. First, on the most general level, language is a resource for constructing and negotiating perspectives on bodily experiences, which then feeds back in the experience of the body. Consequently, there is a “constitutive relationship between language and experience,” as accounts of bodily experiences, such as illness, are “not simply appropriated from available linguistic resources, but physically experienced within these culturally and historically defined boundaries” (Gillies et al., 2004: 110). According to Atkins and Harvey (2010), “our experiences of health and illness are not simply based in the biological “realities” of our bodies, but, crucially, in the language we use to talk about them” (p. 605). More specifically, the language around pain, and its underlying assumptions, also play a key role in health communication. For instance, pessimistic societal beliefs about chronic pain and pessimistic communication from healthcare professionals incite pessimistic beliefs about their illness in pain patients, which then increases levels of disability (Lin et al., 2013; O’Sullivan et al., 2016).

## Methodology

### Data collection

This study is based on 13 clinical encounters between three pain clinic doctors (trained as anaesthesiologists), and 13 chronic pain patients, collected in April-May 2019 at the Ghent University Hospital Pain Clinic (Belgium). All patients have been admitted to the clinic and have seen a pain clinic doctor at least once before. The patients’ age, gender, duration of treatment and kind of pain diagnosis are diverse, as Table 1 below explores.

**Table 1.** Patients’ background and treatment trajectory.

		Diagnosis	Pain clinic since	Gender	Age	Doctor
1	P3	Neuropathic, radicular pain	2012	F	83	DR1
2	P5	Fibromyalgia	2011	F	57	DR2
3	P8	Perineal pain/urinary retention	2018	M	40	DR2
4	P1	Neuropathic, radicular pain	2012	M	70	DR3
5	P2	Fibromyalgia	2016	M	57	DR2
6	P4	Fibromyalgia	2011	F	56	DR2
7	P6	Lower back pain	2014	F	60	DR1
8	P7	Failed back surgery syndrome, neuropathic and radicular pain	2017	F	75	DR3
9	P9	Lower back pain	2011	F	64	DR1
10	P12	Fibromyalgia, joint pain	2012	F	37	DR3
11	P16	Meralgia paresthetica, neuropathic pain	2018	F	44	DR1
12	P20	General pain	2019	F	28	DR1
13	P23	Failed back surgery syndrome	2012	M	57	DR3

The course of and topics addressed during the consultations are varied, as pain patients usually have complex medical histories and interacting problems. They often see different health professionals simultaneously; consequently, the physicians and patients also discuss visits to other health professionals. In general, the patient and physician first talk about how the patient is doing and the issues for which they have planned a consultation, and discuss results from recent treatments or tests. When relevant, a clinical exam is conducted. Consultations furthermore can also consist of (further) diagnostic work and (possible) explanations of new or recently worsened symptoms, discussions of current and new treatments and options for further diagnostic testing, and practical aspects.

Numbers instead of names are used to protect participants’ privacy. The study was approved by the Ghent University Hospital Ethics Review Committee, and all patients gave written consent for their consultations to be recorded and analysed. The researcher was present during the consultations.^1^

### Context

The approach of the pain clinic under scrutiny is BPS-modelled and multidisciplinary: the clinic has an in-house physiotherapist and psychologist. However, admission in the clinic is only done by doctors (in training), and treatment is always first started with doctors/focusing on the biological. When doctors see the potential for increasing quality of life with psychosocial treatment, they discuss patients’ cases with the team, and when deemed relevant, patients also start treatment with the physiotherapist and psychologist. This means that although the general approach of the pain clinic is BPS-modelled, that (1) the care is still compartmentalized across traditional disciplinary borders; and that (2) in the consultations, the doctors mainly focus on the biological and only address psychosocial aspects in specific ways: when it is part of updates on current treatments; when discussing medication as part of psychological care such as antidepressants; and when doctors indicate that they believe psychosocial treatment would be beneficial and explain options and discuss referrals. Occasionally, patients bring up issues with psychosocial aspects on pain themselves. Finally, (3) if doctors mention the possibility of psychosocial care, they are the (first) health professionals in the pain clinic to encounter the patients’ potentially clashing perspectives on the body, and the ones to deal with these.

### Analytical methods and framework

All consultations were transcribed (for transcription conventions, see appendix) and checked for stretches of talk that related to (the interplay of) physical, psychological and social aspects of pain, or the illness experience of the patient. More specifically, these extracts concern the relation between pain and one’s psychological state, such as depression, stress, character traits, fatigue, family life, work, the relation between activities of daily living (ADL) and pain,^2^ and (potential) psychosocial care. First, a general mapping was made of how these topics were discussed, which will be presented in the analysis. The analysis then further focused on extracts of three consultations in which doctor and patients talked about psychological aspects. These extracts were chosen because they illustrate the complexity and diversity of how psychological aspects are discussed, how they relate to assumptions on the body and pain, and how this can lead to misalignment, which is further discussed in the next paragraphs.

Misalignment and disagreements are often not voiced explicitly in interaction in general, and also more specifically in institutional interactions, as it is too face-threatening to do so (Aronsson and Rindstedt, 2011; Lopez-Ozieblo, 2018; Stivers, 2007). For medical interactions, research has found important more implicit indicators are pauses, delays, hesitancies (Stivers, 2007), minimal feedback and minimal or non-committal responses (Aronsson and Rindstedt, 2011), *sotto voce*, low volume, pauses, outdrawn responses, and other prosodic devices (Aronsson and Sätterlund-Larsson, 1987). Similarly, Lopez-Ozieblo (2018) found that in classrooms, forms of mitigated disagreement such as hedging, hesitations, seeking common ground, laughing, and silence are the most used forms of disagreements.

However, other research found that when there is an incongruence between patient and doctor, patients respond more extensively (Heath, 1992), and that when patients disagree on a diagnosis presented by a doctor, they offer additional symptom descriptions or alternative diagnoses (Peräkylä, 2002). Ijäs-Kallio et al. (2010) similarly looked at diagnosis delivery and found that, when patients disagree, they also offer new information, sometimes even after initially aligning with the doctor through a conventional reception token. This new information often draws on further explanations of “immediate symptoms, past experiences with similar illness conditions, and information they have received during past medical visits” (p. 509).

This paper therefore takes a twofold approach to the analysis: (1) it examines interactional devices that can index disagreement and misalignment, as listed above in the reviewed literature and (2) when relevant, the information, arguments and linguistic and interactional resources patients use to explain and account for why they disagree.

## Analysis

### Mapping the 13 consultations

All consultations contained discussions of physical aspects of chronic pain; most of the consultation time is dedicated to discussing symptoms, treatment, risks and side effects. In 3 of the 13 consultations, only physical aspects of the illness experience, medication or other physical treatments were discussed. In 6 consultations, physical and psychological aspects were discussed; in 4 consultations, physical, psychological and social aspects such as work and family life were addressed. In these 4, psychological and social aspects were usually discussed in tandem, often focusing on work or family life as a cause of stress.

When interpreting these numbers, it is important to consider that almost all patients have been in treatment at the pain clinic for a long time, and that the consultations are sometimes very specifically zooming in on one aspect of treatment that needs optimization. In the first three cases, the psychosocial may have been discussed in previous consultations.

In the 10 consultations in which the psychological was a topic, 2 patients clearly and unambiguously aligned with the doctor’s perspective on the interplay between their pain and psychological aspects, and/or the relevance of psychosocial treatment. In 7 cases, the patients did not align with the doctor, ranging from non-committal responses, to ambiguous, and contradictory positioning towards the role of the psychological, to different forms of more explicit resistance. In 1 consultation, the patient was acutely suicidal, which prompted a discussion on immediate psychiatric care, in which the category of misalignment was not relevant to the interaction in the same way as the other 9 of the 10 consultations in which the psychological is discussed. In what follows, the in-depth analysis of three cases is presented.

### Case 1: Consistent dualism and explicit disagreement

In this consultation, the doctor is seeing a patient with perineal pain and urinary retention. The patient has seen many different urologists since his symptoms started. Recently, he visited another urologist who proposed to replace his bladder with a synthetic bladder, which is impactful surgery. The doctor suggests the patient could first consider going to a psychologist.

(1)

1 DR1 maar (.) als ik dat zo hoor dat zijn redelijk drastische dingen

  *but (.) from what I hear those are pretty drastic things*

2 P8 ja ik vind het ook

  *yes I think so too*

3 DR1 en ik heb u vorige keer gezegd ge zou misschien een keer naar de pyscholoog kunnen gaan

  *and I told you last time you could maybe go one time to a psychologist*

4 P8 da da herinner hahahaha ik mij ook ma

  *that that I remember too hahahaha but*

5 DR1 als je die twee nu ten opzichte van elkaar vergelijkt (.)

  *if you compare those two now to one another (.)*

6 P8 ja maar ja het ene is ingrijpend maar het andere niet hé maar de psych- de psycholoog

  *yes but yes the one is impactful/intervening but the other isn’t right but the psych- the psychologist*

7  behalve mij mentaal misschien een boostje geven gaat dat mijn fysiek probleem niet [verbeteren]

  *except for maybe giving me a mental boost.DIM that will not improve my physical [problem]*

In line 1, the doctor assesses the synthetic bladder as drastic, to which the patient explicitly agrees (l2), creating initial alignment between the doctor and patient. In line 3, the doctor proposes that he could also go see a psychologist. The utterance starts with “I told you last time,” which indicates the doctor reopens the discussion of a topic they have addressed before, which may indicate awareness that he is creating a somewhat face-threatening situation. The patient immediately acknowledges he remembers (l4). By thematizing the remembering, the response is drawn out (Aronsson and Sätterlund-Larsson, 1987), and an immediate response to doctor’s proposal is avoided, which is a first marker of misalignment. The utterance is also accompanied by laughter, which is also associated with mitigating disagreement (Lopez-Ozieblo, 2018; Warner-Garcia, 2014). The doctor picks up on this and starts explaining why it is relevant to pursue psychotherapy. The patient interrupts, to actually fully develop the comparison, making use of the contrastive “but” (*maar*) to indicate he will take a different argumentative direction than the doctor was developing. He assesses the surgery as “impactful” (*ingrijpend*), in contrast with the psychologist *not* being so (l6): the psychologist may only give him “a mental (mini-)boost”, but will not improve his physical problem (l7).

This first section shows how the patient’s strong dualistic thinking leads to misalignment between doctor and patient, as the patient explicitly asserts he does not see value in psychotherapy. To account for this assessment, he uses the word “boost” in a diminutive form, suggesting there is room for improvement with regard to his psychological state, but constructing this as marginal and, more importantly, as unrelated to his physical problems.

The doctor and patient go on further discussing the role of stress:

(2)

8 DR1 ge weet dat ze den indruk hebben dat je veel te gestresseerd [zijt]

  *you know they have the impression you are way too [stressed]*

9 P [ja maar] pff misschien wel maar (1.5) allez ik ik ken mijn eigen lichaam (2.0) en

  *[yes but] pff maybe yes but (1.5) well I know my own body (2.0) and*

10  iederen dag gestresseerd mijn pa is ook nen nerveuze gast hij is 73 is nog keihard aan het werken

  *every day stressed my dad also is a nervous bloke he is 73 and still working super hard*

11  kan geen minuut stil blijven maar alé euh als als als het niet gaat gaat het niet hé

  *can’t sit still for a minute but well uhm if if if things are not okay they’re not are they*

12  en en het is niet door een keer te babbelen en een keer te [wenen]

  *and and it is not by having a chat once and [cry once]*

  ((8 turns omitted; doctor reads file and says patient reported before that he does not want to learn relaxation techniques))

21 DR1 ((reading from file)) maar moet wel beroep doen op Xanax en CBD

  *but does have to make use of Xanax and CBD*

22 P ((hoest)) ja omdat dat helpt ((lacht)) en een babbel niet

  *((coughs)) yes because that helps ((laughs)) and a chat doesn’t*

First, the doctor misaligns with the patient’s assessment that he would not benefit from psychotherapy, constructing his level of stress as higher than the patient does, and as too high. He uses the main clause “you know,” with emphasis on “know”. This can have two functions: it can mitigate disagreement by creating common ground as (Lopez-Ozieblo, 2018) as he points to shared knowledge, but it can also challenge the patient’s assessment: of his stress levels as such, but also that he had no prior knowledge of the pain clinic’s assessments of his stress levels. He does so using referring to the “impression” that an unspecified “they” had, which likely refers to the physiotherapist and psychologist of the pain clinic. The doctor here uses the authority of and consensus among his colleagues as a resource to argue that the patient’s stress levels needs to be addressed, which further strengthens the misalignment on the patient’s stress levels.

The patient then partially aligns with the doctor, saying that it “maybe” is the case that he is too stressed, but then says he “knows his body” (l9), using “but” to contrast this initial partial alignment. He accounts for this different assessment, including a number of longer pauses, further signalling potential misalignment and interactional delicacy. His account is built around his experiential knowledge of his illness, which acts as a resource to overrule the assessment of the medical team. Interestingly, his assessment as such is dualistic, but at the same time, he refers to stress as something bodily. More specifically, he explains his high levels of stress as a problem that his dad has too, with which he seems to suggest he either genetically inherited from his father, or that it is the result of his upbringing (l9-10). In doing so, he implies that his stress and urinary problems are unrelated, as the stress has been an issue for much longer than the urinary problems. Moreover, he also appeals to the hierarchy associated with mind-body dualism, more specifically with the evaluative component that psychological problems are usually considered more intentional, and therefore also more problematic. By constructing his stress as familial, he constructs it as an issue that is unintentional and beyond his control, and consequently, he creates another resources to overturn the doctor’s assessment.

When he does so, the doctor remains silent, not using response tokens that are usually associated with aligning when someone talk for a longer stretch of time, despite a number of longer silences (l9). The patient then concludes his argumentation by saying that “crying and talking*”* will not help (l12). These words imply a reductive, negatively connotated representation of psychological treatment, which further advances his assessment of psychological help as having little value for him, and thus now fully resisting the doctor’s assessment of his situation.

The doctor does not respond to this argumentation, but brings up that the patient has indicated before he is not willing to learn relaxation techniques. The doctor challenges this choice by saying that P8 takes calming medication (l21); a final argument that the patient suffers from exceptionally high and problematic levels of stress that need treatment. The patient agrees to the proposition about medication, but renegotiates its meaning: he does not construct medication as (evidence of) a problem, but as an effective solution (l22). This is again accompanied by coping laughter to mitigate disagreement. By renegotiating the meaning of the medication, he again refutes the doctor’s presuppositions about stress and his illness.

These extracts thus clearly revolve around negotiating, refuting and resisting two opposite perspectives on the body and mind, in which doctor and patient are misaligned because the patient consistently refutes a biopsychosocial approach to his illness, as it clashes with his dualistic perspective on his pain. In the following case, a more ambiguous course is taken throughout the consultation.

### Case 2: Alignment on stress, misalignment on treatment options

The following fragments are taken from a consultation with an 83-year-old patient who has been suffering from lower back pain for most of her life. The consultation starts as the patient, her daughter and doctor first discuss the patient has last seen by a pain clinic doctor in 2017. The discussion transitions to the fact the patient has a difficult time managing her activities of daily living, because of her perfectionism: she regularly undertakes big cleaning projects that leave her in intense pain. Both the daughter and the patient herself agree on the fact that this regularly happens. The first twenty minutes of the consultations are mainly dedicated to discussing her perfectionism and its effect on the pain.

The patient then brings up she recently spent some time in a psychiatric hospital because of depression. The doctor and patient discuss the relation between her psychological state and pain, and the doctor says that feelings of depression and anxiety negatively affect the pain. The patient aligns with this, saying the following:

(3)

1 P3 van de stress kan ik dan ook rugpijn hebben

  *of the stress I can have back pain too*

2 DR2 ja en [vice versa]

  *yes and vice versa*

3 P3 [als ik veel stress] heb heb ik ook [veel pijn]

  *if I have a lot of stress I also have a lot of pain*

4 DR2 [ah tuurlijk ja] dat is logisch

  *ah of course yes that’s logical*

5 P3 dat dat is raar hé allez dat is in uwen kop en toch zit dat in uw rug zeg euh het zit het gebeurt allemaal in uw hoofd he

  *that that’s weird huh I mean it’s in your head and still it is in your back say uhm it happens all in your head doesn’t it*

6 DR2 ja het gebeurt allemaal in uw hoofd

  *yes it happens all in your head*

7 P3 alles gebeurt in uw hoofd maar dat dat dan zo’n uitstralingen geeft dat daar zoveel pijn van kunt hebben

  *everything happens in your head but that that then gives such spreading, that you can have so much pain because of that*

This patient aligns with the doctor by saying that her back pain is worsened by stress (l1), constructing a link between her psychological state and her pain. The doctor aligns and confirms this, and adds this connection also works the other way (l2). The patient repeats her initial statement (l3), to which the doctor responds more extensively, constructing the link between her psychological state and physical symptoms as obvious, through adverb “of course,” and the assessment that this link is “logical ***”***. The patient, however, refutes the proposition that this link is obvious, but saying it is “weird” that it is in your head and back, and that what happens in your head “gives such spreading” (l5, l7). This insight thus is constructed as new to the patient.

The patient and doctor disagree on the novelty of this fact, but do align with regard to the role of stress. The patient, as a result of experiential knowledge she gathered, has reconsidered her perspective on her body and her pain, including on stress.

However, as mentioned earlier, another prominent topic in the consultation is the patient’s perfectionist character, and the problematic effect this has on managing her ADL. Both she and her daughter recognize this and tell anecdotes of how she ended up being exhausted and in pain after hours of household work, but does not want to change her ADL. The doctor tells her several times managing ADL (“*doseren*” in Dutch in these extracts) is essential, and explicitly instructs and even warns her to adapt this. And although the patient clearly aligns with the assessment of her character, she does not seem to align with the suggestion to adapt her management of ADL. Throughout the consultation, the patient repeatedly steers the conversation to the topic of medication, and implies a few times that she made the appointment because she hopes to receive a new pill or injection. When the topic has come up a few times, the doctor starts explaining that there are often little to no benefits to medication, and many risks, and managing ADL is always harmless and possibly effective:

(4)

1  dus ge kunt er (.) [niks verkeerd mee doen]

  *so you can’t do anything wrong*

2 P3 ((sighs))

3 DR2 ik weet ‘t is heel moeilijk (.) maar ik ga hetzelfde zeggen of in 2017 (.) ik kan nu daar niet veel aan doen

  *I know it’s very difficult (.) but I’ll tell you the same as in 2017 (.)*

  *I cannot do much about this now*

4 P3 ik denk zelf tegen mij (.) al was ‘t maar voor 6 maanden of [2 maanden]

  *I think to myself (.) if it even was for only 6 months or [2 months]*

5 Daughter [nee nee]

  *[no no]*

6 P3 dat ik een spuitje krijg [dat dat een keer]

  That I get an injection [that I once]

7 Daughter [ik ze ze ze hoopte op een dinge]

  *[I she she was hoping on a thing]*

8 P3 [ja ge hoopt op]

  *[yes you’re hoping for]*

9 DR2 [ja]

  *[yes]*

10 P3 op een spuitje of iets dat ik toch ‘n keer die zeer kwijt was en dat dan ik een keer [kon]

  *for an injection or something that just once I would lose that pain and that I [could]*

11 DR2 [nee]

  *[no]*

12 P3 denken allez

  *think you know*

13 Daughter nee maar als je die [spuit kwijt is was jij]

  *no but if you lose that injection then you*

14 DR2 [zo’n zo’n wonder] zo’n wondermiddelen bestaan niet (.) I am sorry maar dat dat is dr niet (.)

  *such such magic solution does not exist (.) I am sorry but that that isn’t available*

While the doctor explains that managing ADL is a more productive way to deal with her pain than medication or clinical treatment, the patient does not respond or use response tokens. She sighs when the doctor finishes (l2), indicating misalignment. The doctor says he understands this is difficult, which may be a way to realign with the patient. In lines 7–15 however, after several implicit tries, the patient launches a first fully explicit proposal to get an injection, and adds that even short, temporary relief of her pain would be valuable to her. The doctor use one response token, “yes” (l12), but here seems to respond to the daughter’s addition; the doctor then clearly says “no” to the patient (l14), saying that such magical solutions do not exist. He adds an apology and draws out his response (Aronsson and Sätterlund-Larsson, 1987). Beyond this extract, the negotiation continues: P3 also asks for a spinal pain pump and for a photo or scan.

The patient thus complexly aligns and misaligns with the doctor. Although she recognizes that stress and managing ADL affect her pain levels, she came to the consultation with a specific request that is focused entirely on the physical and on medicinal solutions. The perspectives underlying these (mis)alignments are dynamic: her perspective is not fully dualistic, not fully biopsychosocial, but somewhere in between.

### Case 3: From implicit, dualistic non-commitment to more alignment on rumination

In the last case, a similarly dynamic course is taken, but in the other direction. P5 is a patient suffering from fibromyalgia, neuropathic and radicular pain. This section examines how P5 talks to her doctor about recommendations made by other health professionals, but also looks into how the patient brings in the social aspect of her illness by mentioning family circumstances.

Like most other consultations in the data set, the doctor and patient first discuss how the patient is doing, and recent medical visits. The patient tells the pain doctor that she has seen another doctor who proposed to follow a workshop on pain acceptance:

(5)

1 P5 hij stelde voor voor eh in ((ziekenhuis))

  *he proposed to to uhm in ((hospital))*

2  een cursus te volgen dat zou twee keer per week zijn

  *follow a workshop which would be two times a week*

3 DR2 (1) ja

  *(1) yes*

4 P5 den dinsdag en den donderdag om dus eh ja te leren omgaan met pijn en

  *every Tuesday and Thursday to so uhm yes learn how to deal with pain and*

  dat is in samenwerking met psychologen

  *it’s a collaboration with psychologists*

  ((*4 turns omitted*; patient and doctor reconstruct when she went to this doctor))

9 P5 met met dokter ((naam)) had dat gezegd dat ((ziekenhuis)) daarrond werkt

  *with with doctor ((name)) had said that ((hospital)) works on that*

10 DR2 ja

  *yes*

10 P5 rond euh (2) dusja ben daar nu voor ingeschreven

*  on uhm (2) so yes I am enrolled in that now*

11  (2.5) ((doctor typing))

12  maar ja het staat duidelijk ook op de papieren het is niet de bedoeling voor de pijn weg te hebben dus ja (.)

*  but yes it clearly yes on the documents that it is not meant to take away the pain (.)*

13  ik weet al op voorhand dat nie- ((lacht))

  *I already know that not ((laughs))*

14 DR2 (5.5) ((typing)) en dat is waarschijnlijk in groep he

  *and that is probably is a group thing isn’t it*

The patient talks about the workshop, telling that her participation is initiated by the other doctor she has seen, putting herself in the passive, receiving position of the doctor’s active proposing (l1). She talks about the workshop using the epistemic modal *zou* (would) in line 2, indicating it is possible that she will follow the workshop, but not certain. Her account contains a number of hesitations and somewhat longer pauses, in which she seems to wait for the doctor’s assessment or response to the workshop idea. She also explicitly addresses that the workshop is a collaboration with psychologists, addressing the medical professionals and authorities relevant here (l5). After discussing the events’ timeline (l5-8), the patient resumes her account saying that a doctor had told her that the hospital organises these workshops, again referring to a medical authority. She discusses her enrolment using a passive construction, and a long pause (l10). Throughout this section, she thus constructs her linguistic agency (Darics and Koller, 2019) and authority in the matter as low, and she seems to distance herself from the other doctor’s suggestion to participate in the workshop.

She then explicitly voices her doubts, cued by the contrastively used “but” (*maar*), saying that the documents –referring to another (form of) authority- that the workshop will not take away her pain, but focuses on pain management and acceptance. She does not finish her sentence in line 13, but seems to want to express she does not expect much from the workshop, as the negation and laughter indicate. There is a long silence during which the doctor is typing notes; he does not respond to this assessment of the patient, asks about practical details, and then starts discussing medication.

In this extract, the patient initiates a discussion on the value of exploring a new therapy that exclusively lies in the psychosocial realm, and immediately expresses low agency in and low commitment to follow the therapy. She does so to the point she seems to express this therapy will not help her, which she repairs by abandoning her utterance. She may have wanted the doctor to negatively assess the therapy too, overruling the authority of the other doctor and the documents, which he does not- instead he asks about practical details. The doctor does not align with her; his minimal responses indicate he does not pick up on/decides not to react to the implicit negative assessment of the patient. She thus challenges the value of a psychosocial approach for her pain, although not refuting it in such an up-front way as P8. Interestingly, this does not lead to explicit disagreement by the doctor, but he does use the misalignment strategies of non-response and non-commitment, and moving to another topic.

A final relevant extract takes place at the end of the consultation. The patient reflects on the relation between her pain and her husband being ill, breaking a silence while the doctor is typing up notes. The patient suddenly mentions her husband’s cancer has gotten worse. She says that this is “not helping,” to which the doctor responds:

(6)

5 DR2 en als je dan (.) euh neerligt (.) allez ja

*  and if you then lie down then I mean*

6 P5 maar nee nee ik moet zeggen allez (.) eigenlijk dat piekeren euh ik val redelijk goed (1)

*  but no no I have to say I mean actually that brooding um I fall pretty well*

7 DR2 in slaap

*  asleep*

8 P5 als ik mijn trazodone

  *if I my trazodone*

9 Dr [kan pakken dan gaat (xxx)]

  [can take then it goes (xxx)}

10 P5 ‘k had gedacht dat dat veel slechter zou geweest zijn

*  if I my trazodone I thought that that would be much worse*

11 Dr ja

*  yes*

12  maar euh het is vooral van de pijn dat ik niet in slaap val

*  but uhm it is mainly because of the pain that I do not fall asleep*

13 DR2 ja

*  yes*

14 P5 want die pijn overheerst precies gelik mijn pie-piekergedachten

*  because that pain dominates my negative thoughts*

15 DR2 ja

*  yes*

16 P5 het is niet dat ik mij kom allez ik heb precies gelik teveel pijn voor te kunnen piekeren

*  it’s not that I me come I mean I seem to have too much pain to be able to brood*

17  ik ga het zo zeggen (0.5) maar eh het is natuurlijk in bed allez

*  I’ll say it like this but uhm it’s of course in bed I mean*

  ((doctor breathes out audibly and types))

18  ik word wakker ‘s nachts om te plassen en ((voice gets shaky))

  dat komt in mijn hoofd

*  I wake up at night to go pee and then it comes into my head*

19  ((starts crying))

20  dus het moet zijn

*  so it has to be*

21 DR2 ja

*  yes*

22 P5 dat ik ermee bezig ben he ((huilt))

*  that I am concerned with it ((cries))*

She first constructs the impact of her husband’s condition on her wellbeing as evident, by bringing it up during a silence, and by using the word “of course” (l1)—it remains unclear, however, whether she is talking about the impact on pain and/or on her wellbeing or psychological state. The doctor follows up on this suggesting her husband’s situation keeps her awake (l5), with pauses and hesitation markers, indicating he may see this proposition as delicate. She refutes this with a double negation (“but no no”), and argues her pain is the primary factor affecting her sleep, saying that the (physical) pain makes it impossible to ruminate (l12-14). However, she then rebuts this, saying she ruminates when she wakes up at night, which she causally links through “so” to the fact that it does affect her sleep, while crying.

She thus is more ambiguous about psychological and social aspects in this extract than before; she does not directly link them to her pain, but does bring them up spontaneously while the doctor is typing. She constructs pain and rumination as separate, and pain as primary and stronger than rumination. However, she concludes that her worries do affect her sleep. This extract shows again that perspectives on the relation between mind, body and the social are dynamic, causing rapidly changing dynamics of alignment and misalignment within one consultation, and even within the discussion of one particular aspect.

## Discussion and conclusion

This paper presents an analysis of the misalignment and resistance that results from differing perspectives on bodily experiences in consultations in a pain clinic taking a biopsychosocial approach to treatment. The analysis shows that perspectives on and relations between body, mind, and social dimensions indeed are intensely negotiated in these consultations, and that patients construct a range of more or less dualistic understandings that are not compatible with the doctors’ BPS approach. For instance, P8 sees no connection between his physical pain and stress; he sees pain as a purely physical issue. This causes misalignment and disagreement when the doctor suggests seeing a psychotherapist, which remains unsolved throughout the consultation. P3 sees connections between stress and pain initially, aligning with the doctor. However, it then becomes clear that patient and doctor have different perspectives on what could ease her pain—the doctor focuses on ADL and perfectionism, while the patient wants a physical treatment, such as an injection–, creating misalignment to the point the doctor tells her he cannot help her as she wishes. P5 is navigating somewhere in between, in more implicit ways: she sees no value in psychosocially-oriented therapy, causing minimal responses on the side of the doctor, but eventually aligns with the doctor regarding how her sleeping problems are not just related to the pain, but also negatively impacted by her husband’s cancer prospects and treatments.

Consequently, categorizing patients as purely constructing dualistic or biopsychosocial perspectives is not possible, as patients dynamically construct different perspectives on the interplay of the physical, psychological and social, within one consultation. In the cases discussed, the differing perspectives leading to misalignment come with a range of argumentative resources on both the doctors’ and the patients’ side. Perhaps most importantly, the analysis shows a relation between differing perspectives on the body and illness, and how patients react to certain proposals for treatments. When patients do not see a relation between pain and psychosocial factors, they may negatively evaluate and sometimes dismiss (the value of) certain treatment options.

In sum, this paper illustrates that, for physicians, integrating models such as the biopsychosocial model into clinical practice is complex (Dwyer et al., 2017; Purcell et al., 2019). In the context of this paper, it is complex for the pain clinic’s physicians to do so because of their disciplinary background and training, as discussed earlier, and because of how the pain clinic is set up, with still relatively strong disciplinary boundaries between the doctors on the one hand, and physiotherapist and psychologist on the other. This set-up not only influences the context doctors work in, but also may also prime patients to expect a mainly or exclusively biological focus, which can affect their expectations of the consultation and treatment options. However, the analysis also indicates there are other crucial factors, such the complex interplay between patient expectations more broadly, their dynamic understanding of their pain, (dualistic) societal perspectives on pain, and providers’ resources—argumentatively and treatment-wise. These aspects can be seen as aspects of culture; which indicates that especially culture as a sub-element of the social domain, which also covers aspects such as interpersonal relations, work history and environmental stressors, indeed deserves further development in the BPS model (Benning, 2015). The relation between sociocultural assumptions on the body and illness on the one hand, and social expectations, both within health care communication, and beyond, on the other, must be emphasized and developed more. Moreover, it is crucial that the heterogeneity and dynamic nature of these assumptions and expectations are highlighted more, as also argued by Benning (2015) and Hatala (2012).

As the implementation of the BPS model in practice can be difficult, this paper wants to propose a tentative recommendation for both research and clinical practice. The data indicates that sociocultural assumptions on the body affect health care communication, but also that these perspectives are not stable, and clearly dichotomous, but dynamic and multi-layered. I propose to see dualistic and biopsychosocial perspectives in communication not as two categories, but as poles of a continuum, on which patients and providers dynamically shift across and within interactions, further extending Jull’s (2017) proposal to see the domains of the BPS models as fluid. This is important for further research, but may also be a useful perspective for health professionals.

This brings us to limitations and areas for further research. This study is exploratory in nature and relatively small in its set-up; consequently, the findings are context-dependent as they concern only 13 consultations, collected in one pain clinic, in one specific cultural and social context. At the same time, due to limitations of scope, a number of social and cultural factors, such as gender, race, age, socio-economic background and specific pathology, were not taken into account, while potentially being influential. Consequently, more research is needed to look into the impact of the factors mentioned above. Moreover, chronic pain is not only an issue in tertiary care, and contexts like primary care and physiotherapy provide equally important sites for talk about chronic pain. In the same vein, more comparative work with a focus on different types of clinicians in multidisciplinary pain care contexts can further shed light on the co-constructive nature of constructing perspectives on the body, mind, and pain.

Finally, the operationalisation of the factors of the BPS model is relatively simple. More research is needed on specific discursive aspects of constructing the body (and mind) in clinical interactions, with more specific attention to subcategories of the BPS model, and the evaluations with which these constructs come, especially with regard to taboo and stigma. From a discourse perspective, more is possible with other discursive analytical categories such as metaphors, and actors and agency, and so on.

## Appendix

**Appendix.** Transcription conventions.

(.)	micropause
(number)	longer pause, number of seconds between brackets
[ ]	overlap
(( ))	non-verbal sounds, omissions, other features of the interaction
(word)	words/information omitted because of privacy concerns
(xxx)	Incomprehensible/inaudible
.dim	diminutive

## Notes

1. As with all observational qualitative research, but specifically in context with researchers present, this requires some more reflection on participant reactivity (Paradis and Sutkin, 2017). A number of steps were taken to reduce it to a minimum: to ask patients whether they wanted to participate, they were first called by the secretary of the clinic. When they were interested, they were invited to come to their consultation early, so that the author of the paper could have a 10 to 20-minute conversation with the potential participants to discuss and sign the informed consent form. This also allowed for getting somewhat familiar with the participants, to reduce participant reactivity. A second measure taken was that the IC forms only contained a general research question/topic, and did not specifically address the researcher’s interest in mind-body dualism. Third, only audio recordings were made, to reduce the intrusiveness of the data collection. The researcher was present during the consultation, and took notes of times when participants shifted their gaze at her. This happened only very occasionally. There were no indications of strong participant reactivity.

2. ADL is an important concept in treating chronic pain, as it can be hard to find out what brings the best quality of life for patients in terms of ADL. Some activities that are unproblematic for healthy people can easily become too strenuous for chronic pain patients and worsen their pain, but they also need to try to keep moving and to keep having goals. Even when it is technically (more or less) clear for a patient how to strike that balance, it can remain hard to accept what is possible, and what is not, and to adhere to set goals.
